# Clinical use of Bevacizumab in treating refractory glaucoma

**Published:** 2015

**Authors:** V Popescu, S Pricopie, M Totir, R Iancu, S Yasyn, C Alexandrescu

**Affiliations:** * Anatomy Department, “Carol Davila” University of Medicine and Pharmacy, Bucharest, Romania; ** Department of Clinical Ophthalmology, University Emergency Hospital Bucharest, Romania; *** Ophthalmology Department, Regina Maria - private health care network, Bucharest, Romania; **** Ophthalmology Department, “Carol Davila” University of Medicine and Pharmacy, Bucharest, Romania

**Keywords:** bevacizumab, glaucoma, trabeculectomy, vascular endothelial growth factor

## Abstract

Bevacizumab is a recombinant humanized monoclonal antibody directed against vascular endothelial growth factor (VEGF). Though other VEGF inhibitors are being approved for the treatment of ophthalmological conditions, bevacizumab found its way into ophthalmology and clinical practice all around the world.

The objective of this review is to present the ophthalmic dosage and administration pathways of bevacizumab in treating refractory glaucoma patients.

**Abbreviations:** VEGF = vascular endothelial growth factor, FDA = Food and Drug Administration, AMD = age-related macular degeneration

## Introduction

Medications, such as ranibizumab, pegaptanib, alfibercept and bevacizumab, action by inhibiting vascular endothelial growth factor (VEGF). As a consequence, they inhibit angiogenesis. Although only pegaptanib and ranibizumab are approved for treatment of ophthalmological conditions, bevacizumab is also used in ophthalmology as an off-label drug since 2004 [**1**]. Bevacizumab is Food and Drug Administration (FDA) approved for the treatment of colorectal cancer [**2**,**3**]. Despite its off-label use, bevacizumab is the most widely used anti-VEGF factor in ophthalmology [**4**,**5**].

**Mechanism of Action and Pharmacokinetics**

In 1971, Judah Folkman reported in the “New England Journal of Medicine” that all cancer tumors are angiogenesis-dependent [**6**]; he was the first to use the term "anti-angiogenic therapy" and bevacizumab became the first therapy approved by the US FDA designed to inhibit angiogenesis in tumors [**7**].

VEGF represents an angiogenic inducer in vivo and an endothelial cell-specific mitogen in vitro. VEGF is a dimeric glycoprotein of 36-46 kD which binds on the surface of endothelial cells and initiates endothelial proliferation and the formation of new blood vessels (angiogenesis). This growth factor plays a key role in developmental angiogenesis, being one of the most potent positive regulators, and also demonstrated to act as a mediator of pathological angiogenesis [**8**]. VEGF is a potent mitogen and survival factor for endothelial cells. (VEGF)-A seems to represent the primary target of recent anti-angiogenic strategies.

Bevacizumab (Avastin, Roche) acts by inhibitig the binding of VEGF to its receptors, thus preventing the angiogenesis. Bevacizumab is humanized monoclonal antibody designed against the biologically active isoforms of VEGF-A [**8**]. It is derived from the murine VEGF monoclonal antibody, combining over 90% human protein sequence with about 7% murine protein sequence [**9**]. Bevacizumab has a molecular weight of about 149kD, with structure of recombinant IgG antibody. Bevacizumab is preconditioned as a clear to slightly opalescent, colorless to pale brown, sterile solution with slightly acidic pH of 6.2. The product is formulated in alpha-trehalose dihydrate, sodium phosphate (monobasic, monohydrate), sodium phosphate (dibasic, anhydrous), polysorbate and water for injection.

Bevacizumab has a longer systemic half life, compared with other VEGF inhibitors (e.g. ranibizumab), due to its glycosylated structure [**10**]. Despite the fact that it is not approved for intravitreal use [**11**], it is often used as an off-label drug by ophthalmologists. This anti-cancer drug found its way in ophthalmology and clinical practice all around the world because the costs of the therapy with bevacizumab are much lower than with other similar VEGF inhibitors. Ranibizumab is a humanized antibody fragment (Fab) directed against VEGF-A produced in an E. coli expression system which was specifically designed for intravitreal use and which was approved for use in EU and USA. Although there are several ongoing trials that compare the two medications [**12**,**13**], the differences between the two drugs regarding safety and efficacy are still debatable.

**Clinical Use in Ophthalmology**

The mainstay treatment of exudative form of age-related macular degeneration (AMD), which is one of the most encountered ocular pathologies, is represented by intravitreal injection of anti-VEGF. Despite the fact that there are a couple of anti-VEGF types of drugs currently approved for intraocular use, off-label use of bevacizumab continues to be most widely spread among ophthalmologists.

Although the primary use of bevacizumab in ophthalmology remains the treatment of exudative AMD, a lot of other ocular entities are treated nowadays with this medication (**Table 1**).

**Table 1:** Ocular entities that can be treated with bevacizumab

Ocular pathologies treated with Bevacizumab	
Retinal neovascularization	Proliferative diabetic retinopathy
	Central retinal vein occlusion
	Branch retinal vein occlusion
	Central retinal artery occlusion
	Ocular ischemic syndrome
	Retinopathy of prematurity
	Sickle cell retinopathy
Choroidal neovascularization	Exudative age-related macular degeneration
	Angioid streaks
	Pathologic myopia
	Best disease
	Multifocal choroiditis
	Central serous chorioretinopathy
	Uveitis
Pterygium	
Macular edema	Diabetic retinopathy
	Pseudophakic
	Branch retinal vein occlusion
	Central retinal vein occlusion
	Uveitic
Corneal neovascularization	Corneal graft rejection neovascularization
	Herpetic corneal neovascularization
	Dry eye associated corneal neovascularization
Glaucoma surgery	Adjunct to glaucoma filtering surgery
	Bleb revision
Neovascular glaucoma	

**Ophthalmic dosage and administration**

Bevacizumab can be administered for refractory glaucoma using various pathways: intravitreal, topical, subconjunctival and intracameral.

1. Intravitreal administration

The use of anti-VEGF agents has expanded greatly over lasts years to include corneal neovascularization and neovascular glaucoma. Human clinical studies were performed because the key pathologies of neovascular glaucoma are iris neovascularization and fibrovascular membrane proliferation in the iridocorneal angle. A lot of studies reported a reduction of iris neovascularization and/or reduction of IOP after intravitreal bevacizumab injections in neovascular glaucoma [**14**]. Another study noted complete or partial reduction of leakage in iris fluorescein angiography after intravitreal or intracameral injections of bevacizumab [**15**-**17**]. Subsequently, larger case series reported the potential value of bevacizumab in the treatment of neovascular glaucoma [**18**]. Combined with panretinal photocoagulation, intravitreal bevacizumab produce a faster decrease in IOP [**19**]. Treatment regimen comprises of 1.25 mg/ 0.05 ml bevacizumab injected into vitreal cavity, in single or multiple administrations depending on the clinical response. Clinical trials compare various administration frequencies. Cornish et al. used for the first time bevacizumab as adjunct to trabeculectomy. After intravitreal injection post trabeculectomy with MMC, IOP was maximum 15mmHg without glaucoma medication at 6 month.

2. Topical administration

Topical administration is a potential treatment modality for corneal neovascularization and neovascular glaucoma. There are studies that confirm the efficacy of high dose 25 mg/ ml bevacizumab instillations administered twice daily [**20**,**21**], four or five times daily [**22**]. In eyes with a high risk of failure, topical application might favor normal bleb function, with a significantly lower proportion of bleb leaks. Corneal penetration of bevacizumab was demonstrated to be extremely low even at higher doses and following more prolonged treatment algorithms [**23**-**26**]. If corneal epithelium is intact, it should represent an effective barrier that cannot be penetrated by molecules larger than 1 nm [**27**]; bevacizumab size is approximately 12 nm, therefore it does not reach significant intraocular levels. When epithelial defects are present, bevacizumab can penetrate cornea [**28**] and alters corneal healing process, also causing stromal melting after prolonged use [**29**-**31**].

Considering the above mentioned characteristics and despite several studies that reported safety profiles for topical administration, intraocular penetration may occur after prolonged topical administration of bevacizumab in patients with corneal neovascularization and epithelial defects [**29**,**32**-**34**]. While the median inhibitory concentration in vitro of bevacizumab was found to be 22 ng/ ml, the minimal concentration effectively blocking endothelial cell proliferation induced by VEGF was 500 ng/ ml [**35**]. This result shows that, by topical administration, therapeutic efficacy will be obtained with minimal risks for intraocular or systemic adverse effects [**24**].

3. Subconjunctival administration

Subconjunctival administration can be used for recurrent pterygium, for bleb failure modulation after glaucoma surgery, for corneal neovascularization etc. The dosage ranges between 1.25 mg and 2.5 mg. Nomoto et al demonstrated in his study on rabbit eyes that bevacizumab applied subconjunctivally had a longer half-life than intravitreal administration [**36**]. Grewal used subconjunctival bevacizumab in a pilot study of 12 patients with open and closed angle glaucoma at the end of glaucoma surgery. During surgery, no other fibrotic agent was used. Success, defined as IOP of 6-16 mm Hg without medication, was achieved by 11 patients (92%) at 6 months. Mean IOP decreased to 11.6 mm Hg with no additional medication. Another research team compared the effects of bevacizumab and 5-FU as adjunct to trabeculectomy. They found no significant differences of IOP between the two groups at the end of the study, but medical therapy was needed to obtain successful IOP for more patients in bevacizumab group. On the other hand, adjunctive bevacizumab caused minor reduction in corneal endothelial cell count.

In Choi et al. case series study, were included six eyes with refractory glaucoma which undergone laser or surgical treatment. They underwent trabeculectomy with MMC and received a subconjunctival injection of 1.25 mg bevacizumab at the end of the surgery.

Based on the favorable results of this study, this report suggests that bevacizumab administered subconjunctivally may be effective in improving glaucoma filtration surgery.

The largest prospective randomized double-masked study was realized by Vandewalle et al. This trial investigates the utility of anti-VEGF as adjunct to glaucoma surgery. Bevacizumab was administered as a single intracameral injection at the end of trabeculectomy. The absolute success was significantly higher in bevacizumab group. An important fact is that IOP at the end of the study was similar in bevacizumab and placebo group, but at a cost of more frequent needlings in the placebo group.

4. Intracameral administration

The use of anti-VEGF drugs may be appealing for glaucoma patients because high levels of VEGF were found in their aqueous humor [**37**,**38**]. Intracameral administration (1.25 mg/ 0.05 ml) of bevacizumab is lately used as a replacement or as an adjunctive to antimetabolites for glaucoma filtration surgery. The largest prospective randomized double-masked study was realized by Vandewalle et al. Bevacizumab was administered as a single intracameral injection at the end of trabeculectomy. The absolute success was significantly higher in bevacizumab group. An important fact is that IOP at the end of the study was similar in bevacizumab and placebo group, but at a cost of more frequent needlings in the placebo group [**39**].

## Conclusion

The use of unlicensed treatments in medicine is currently under debate, with wide implications for patient safety and informed consent.

Bevacizumab use in ophthalmology targets a large number of ocular entities as an off-label drug, due to its low cost and good safety profile. Because its multiple ways of administration bevacizumab can be used for the treatment of more than 20 ophthalmic pathologies.

Anti-VEGF therapies may play a role in arresting or reversing the progression of glaucomatous disease, because of their potential inhibitory effect on postoperative new vessels growth and fibroblastic proliferation at the site of the bleb.

Over the past few years, many studies have investigated the utility of anti-VEGF agents in the treatment of NVG and filtering bleb survival and rescue. Investigators have tried different routes of bevacizumab administration with potential ocular effects, including intravitreal injection, intracameral injection, subconjunctival injection and topical administration. Through future research, the antiangiogenic and antifibroblastic properties of anti-VEGF agents may prove to be beneficial in patients being treated for various forms of the blinding condition of glaucoma.

**Acknowledgement**

This paper is partly supported by the Sectorial Operational Programme Human Resources Development (SOPHRD), financed by European Social Found and the Romanian Government under the contract number POSDRU/159/1.5/S/137390.

## References

[1] Shahsuvaryan ML (2013). Avastin: Therapeutic Potential in Vascular Retinopathy due to Retinal Vein Occlusion. Bulletin of Environment. Pharmacology anf Life Sciences.

[2] Hurwitz HI, Fehrenbacher L, Hainsworth JD, Heim W, Berlin J, Holmgren E (2004). Bevacizumab plus irnotecan, fluorouracil and leucovorin for metastatic colorectal cancer. N Engl J Med.

[3] Ferrara N, Hillan KJ, Gerber HP, Novotny W (2004). Discovery and development of bevacizumab, an anti-VEGF antibody for treating cancer. Nat. Rev., Drug Discov.

[4] Lim LS, Mitchell P, Seddon JM, Holz FG, Wong TY (2012). Age-related macular degeneration. Lancet.

[5] Brechner RJ, Rosenfeld PJ, Babish JD, Caplan S (2011). Pharmacotherapy for neovascular age-related macular degeneration: an analysis of the 100% 2008 medicare fee-for-service part B claims file. Am J Ophthalmol.

[6] Folkman J (1971). Tumor Angiogenesis: Therapeutic Implication. New England Journal of Medicine 285.

[7] Grisanti S, Ziemssen F (2007). Bevacizumab: Off-label use in ophthalmology. Indian J Ophthalmol.

[8] Ferrara N, Hillan KJ, Nowotny W (2005). Bevacizumab (Avastin), a humanized anti-VEGF monoclonal antibody for cancer therapy. Biochem Biophys Res Commun.

[9] Presta LG, Chen H, O'Connor SJ, Chislom V, Meng YG, Krummen L, Winkler M, Ferrara N (1997). Humanization of an anti-vascular endothelial growth factor monoclonal antibody for the therapy of solid tumors and other disorders. Cancer Res.

[10] Kim H, Robinson SB, Csaky KG (2009). FcRn receptor-mediated pharmacokinetics of therapeutic IgG in the eye. Mol Vis.

[11] Ferrara N, Hillan KJ, Gerber HP, Novotny W (2004). Discovery and development of bevacizumab, an anti-VEGF antibody for treating cancer. Nat Rev Drug Discov.

[12] Martin DF, Maguire MG, Ying GS, Grunwald JE, Fine SL, Jaffe GJ (2011). Ranibizumab and bevacizumab for neovascular agerelated macular degeneration. N Engl J Med.

[13] Martin DF, Maguire MG, Fine SL, Ying GS, Jaffe GJ, Grunwald JE, Toth C, Redford M, Ferris FL (2012). Ranibizumab and bevacizumab for treatment of neovascular age-related macular degeneration: two-year results. Ophthalmology.

[14] Davidorf FH, Mouser JG, Derick RJ (2006). Rapid improvement of rubeosis iridis from a single bevacizumab (Avastin) injection. Retina.

[15] Grisanti S, Biester S, Peters S, Tatar O, Ziemssen F, Bartz-Schmidt KU (2006). Tuebingen Bevacizumab Study Group. Intracameral bevacizumab for iris rubeosis. Am J Ophthalmol.

[16] Gheith ME, Siam GA, de Barros DS, Garg SJ, Moster MR (2007). Role of intravitreal bevacizumab in neovascular glaucoma. J Ocul Pharmacol Ther.

[17] Chalam KV, Gupta SK, Grover S, Brar VS, Agarwal S (2008). Intracameral Avastin dramatically resolves iris neovascularization and reverses neovascular glaucoma. Eur J Ophthalmol.

[18] Wakabayashi T, Oshima Y, Sakaguchi H, Ikuno Y, Miki A, Gomi F, Otori Y, Kamei M, Kusaka S, Tano Y (2008). Intravitreal bevacizumab to treat iris neovascularization and neovascular glaucoma secondary to ischemic retinal diseases in 41 consecutive cases. Ophthalmology.

[19] Ehlers JP, Spirn MJ, Lam A, Sivalingam A, Samuel MA, Tasman W (2008). Combination intravitreal bevacizumab/ panretinal photocoagulation versus panretinal photocoagulation alone in the treatment of neovascular glaucoma. Retina.

[20] Waisbourd M, Levinger E, Varssano D, Moisseiev E, Zayit-Soudri S, Barak A, Loewenstein A, Barequet I (2013). High-dose topical bevacizumab for corneal neovascularization. Pharmacology.

[21] Moisseiev E, Waisbourd M, Ben-Artsi E, Levinger E, Barak A, Daniels T, Csaky K, Loewenstein A, Barequet IS (2014). Pharmacokinetics of bevacizumab after topical and intravitreal administration in human eyes. Graefes Arch Clin Exp Ophthalmol.

[22] Waisbourd M, Shemesh G, Kurtz S, Rachmiel R, Moisseiev E, Zayit-Soudri S, Loewenstein A, Barequet (2014). Topical bevacizumab for neovascular glaucoma: a pilot study. I.Pharmacology.

[23] Nomoto H, Shiraga F, Kuno N, Kimura E, Fujii S, Shinomiya K, Nugent AK, Hirooka K, Baba T (2009). Pharmacokinetics of bevacizumab after topical, subconjunctival, and intravitreal administration in rabbits. Invest Ophthalmol Vis Sci.

[24] Yoeruek E, Ziemssen F, Henke-Fahle S, Tatar O, Tura A, Grisanti S, Bartz-Schmidt KU, Szurman P (2008). Bevacizumab Study Group Safety, penetration and efficacy of topically applied bevacizumab: evaluation of eyedrops in corneal neovascularization after chemical burn. Arch Ophthalmol.

[25] Dastjerdi MH, Sadrai Z, Saban DR, Zhang Q, Dana R (2001). Corneal penetration of topical and subconjunctival bevacizumab. Invest Ophthalmol Vis Sci.

[26] Kim MJ, Han ES, Kim JW, Kim TW (2010). Aqueous humor concentration of bevacizumab after subconjunctival injection in rabbit. J Ocul Pharmacol Ther.

[27] Prausnitz MR, Noonan JS (1998). Permeability of cornea, sclera, and conjunctiva: a literature analysis for drug delivery to the eye. J Pharm Scil.

[28] Dastjerdi MH, Sadrai Z, Saban DR, Zhang Q, Dana R (2001). Corneal penetration of topical and subconjunctival bevacizumab. Invest Ophthalmol Vis Sci.

[29] Kim SW, Ha BJ, Kim EK, Tchah H, Kim TI (2008). The effect of topical bevacizumab on corneal neovascularization. Ophthalmology.

[30] Galor A, Yoo SH (2010). Corneal melt while using topical bevacizumab eye drops. Ophthalmic Surg Lasers Imaging.

[31] Kim TI, Chung JL, Hong JP, Min K, Seo KY, Kim EK (2009). Bevacizumab application delays epithelial healing in rabbit cornea. Invest Ophthalmol Vis Sci.

[32] Uy HS, Chan PS, Ang RE (2008). Topical bevacizumab and ocular surface neovascularization in patients with Stevens–Johnson syndrome. Cornea.

[33] Dastjerdi MH, Al-Arfaj KM, Nallasamy N, Hamrah P, Jurkunas UV, Pineda R, Pavan-Langston D, Dana R (2009). Topical bevacizumab in the treatment of corneal neovascularization. Arch Ophthalmol.

[34] Koenig Y, Bock F, Horn F, Kruse F, Straub K, Cursiefen C (2009). Short- and long-term safety profile and efficacy of topical bevacizumab (Avastin) eye drops against corneal neovascularization. Graefes Arch Clin Exp Ophthalmol.

[35] Wang Y, Fei D, Vanderlaan M, Song A (2004). Biological activity of bevacizumab, a humanized anti-VEGF antibody in vitro. Angiogenesis.

[36] Nomoto H, Shiraga F, Kuno N, Kimura E, Fujii S, Shinomiya K, Nugent AK, Hirooka K, Baba T (2009). Pharmacokinetics of bevacizumab after topical, subconjunctival, and intravitreal administration in rabbits. Invest Ophthalmol Vis Sci.

[37] Li Z, Van Bergen T, Van de Veire S, Van de Vel I, Moreau H, Dewerchin M, Maudgal PC, Zeyen T, Spileers W, Moons L, Stalmans I (2009). Inhibition of vascular endothelial growth factor reduces scar formation after glaucoma filtration surgery. Invest Ophthalmol Vis Sci.

[38] Tripathi RC, Li J, Tripathi BJ, Chalam KV, Adamis AP (1998). Increased level of vascular endothelial growth factor in aqueous humor of patients with neovascular glaucoma. Ophthalmology.

[39] Vandewalle E, Pinto LA, Bergen TV, Spielberg L, Fieuws S, Moons L, Spileers W, Zeyen T, Stalmans I (2014). Intracameral Bevacizumab as an Adjunct to Trabeculectomy. Br J Ophthalmol.

